# Lung eosinophils elicited during allergic and acute aspergillosis express RORγt and IL-23R but do not require IL-23 for IL-17 production

**DOI:** 10.1371/journal.ppat.1009891

**Published:** 2021-08-31

**Authors:** Bhawna Yadav, Charles A. Specht, Chrono K. Lee, Maria Pokrovskii, Jun R. Huh, Dan R. Littman, Stuart M. Levitz

**Affiliations:** 1 Department of Medicine, University of Massachusetts Medical School, Worcester, Massachusetts, United States of America; 2 The Kimmel Center for Biology and Medicine of the Skirball Institute, New York University School of Medicine, New York, New York, United States of America; 3 Department of Immunology, Blavatnik Institute, Harvard Medical School, Boston, Massachusetts, United States of America; 4 Evergrande Center for Immunologic Diseases, Harvard Medical School and Brigham and Women’s Hospital, Boston, Massachusetts, United States of America; 5 Howard Hughes Medical Institute, New York, New York, United States of America; Memorial Sloan-Kettering Cancer Center, UNITED STATES

## Abstract

Exposure to the mold, *Aspergillus*, is ubiquitous and generally has no adverse consequences in immunocompetent persons. However, invasive and allergic aspergillosis can develop in immunocompromised and atopic individuals, respectively. Previously, we demonstrated that mouse lung eosinophils produce IL-17 in response to stimulation by live conidia and antigens of *A*. *fumigatus*. Here, we utilized murine models of allergic and acute pulmonary aspergillosis to determine the association of IL-23, IL-23R and RORγt with eosinophil IL-17 expression. Following *A*. *fumigatus* stimulation, a population of lung eosinophils expressed RORγt, the master transcription factor for IL-17 regulation. Eosinophil RORγt expression was demonstrated by flow cytometry, confocal microscopy, western blotting and an mCherry reporter mouse. Both nuclear and cytoplasmic localization of RORγt in eosinophils were observed, although the former predominated. A population of lung eosinophils also expressed IL-23R. While expression of IL-23R was positively correlated with expression of RORγt, expression of RORγt and IL-17 was similar when comparing lung eosinophils from *A*. *fumigatus*-challenged wild-type and IL-23p19^-/-^ mice. Thus, in allergic and acute models of pulmonary aspergillosis, lung eosinophils express IL-17, RORγt and IL-23R. However, IL-23 is dispensable for production of IL-17 and RORγt.

## Author summary

Humans regularly inhale spores of *Aspergillus fumigatus*, a common environmental fungus. While such exposure is of little consequence to most, in persons with impaired immune systems it can cause a spectrum of diseases ranging from invasive aspergillosis to allergic aspergillosis. A type of white blood cell called the eosinophil is a defining feature of allergic aspergillosis. Despite their importance, the contribution of eosinophils to this disease state is poorly understood. We previously demonstrated that eosinophils produce the cytokine IL-17 in murine models of aspergillosis models. Here, we defined the contributions of two molecules, the transcription factor RORγt and the cytokine IL-23, to eosinophil IL-17 production. These two molecules are important for optimal IL-17 production in other cell types. We discovered a population of lung eosinophils express RORγt. While expression of the receptor for IL-23 (IL-23R) was positively correlated with expression of RORγt, using mice deficient in IL-23, we showed IL-23 was not required for expression of RORγt and IL-17. Thus, challenge of lungs with live *A*. *fumigatus* or its antigens skews lung eosinophils towards IL-17 production by a pathway that is independent of IL-23. Our results advance our understanding of eosinophil plasticity and have implications for the development of therapeutic approaches for treating allergic lung diseases.

## Introduction

Fungi of the genus *Aspergillus* are ubiquitous in the environment [1]. It is estimated that individuals typically inhale hundreds of conidia a day. While in the immunocompetent host this is generally without consequence, in individuals with dysregulated immune systems, the conidia may germinate into hyphae and cause a wide spectrum of diseases ranging from invasive aspergillosis in severely immunocompromised hosts to allergic aspergillosis in atopic individuals [2]. Allergic aspergillosis comprises an overlapping set of diseases including severe asthma with fungal sensitization, allergic bronchopulmonary aspergillosis (ABPA) and chronic pulmonary aspergillosis. The burden of invasive and allergic aspergillosis is estimated at over 200,000 and 5,000,000 persons per year, respectively [3, 4]. Only a few of the hundreds of described *Aspergillus* species regularly cause human disease with *A*. *fumigatus* being the most common.

Clinical and experimental studies point to critical roles for multiple cell types in orchestrating innate and adaptive host defenses against pulmonary aspergillosis. Consistent with the wide spectrum of manifestations of aspergillosis, the relative importance of each cell type is dependent upon the model of aspergillosis being studied. It is difficult to discern the role of eosinophils in human invasive aspergillosis; while risk factors such as chemotherapy and corticosteroids deplete eosinophils, they also depress other arms of the immune system. However, in chronic cases of human IPA featuring less immunosuppression, eosinophils are frequently found [5]. We and others have examined the contribution of eosinophils in mouse models of aspergillosis. Eosinophils release extracellular traps [6] and have potent antifungal activity in vitro [7]. The susceptibility to acute infection with *A*. *fumigatus* has been studied in mice lacking eosinophils; two studies found increased susceptibility [7, 8], whereas one study found decreased susceptibility [9]. The reasons for the disparity are speculative but could be related to different fungal strains used in the models. Data from a variety of models, including those featuring eosinophil-deficient mice, provide strong evidence that eosinophilic inflammation in the airways is pathogenic in allergic pulmonary inflammation [7, 10, 11].

The antimicrobial and immunoregulatory activity of eosinophils is mediated in large part by effector molecules stored in their primary and secondary granules, including eosinophil peroxidase (EPX) and major basic protein (MBP). Importantly, eosinophils pre-synthesize cytokines and store them in their granules, enabling rapid secretion upon stimulation [12]. Over 35 cytokines, chemokines and growth factors have been identified in at least some eosinophil populations [12, 13].

The IL-17 cytokine family is comprised of IL-17A, IL-17B, IL-17C, IL-17D, IL-17E (also known as IL-25) and IL-17F [14]. IL-17A and IL-17F have the most similarity and exist as disulfide linked homodimers and heterodimers. IL-17 family members mediate their biological activity by binding to receptors for IL-17 (IL-17R), of which there are at least five members [14]. Notably, IL-17A, IL-17F and IL-17AF bind to a heterodimeric complex comprised of IL-17RA and IL-17RC. In this manuscript, IL-17 is used to refer to IL-17A. A preponderance of experimental mouse data and human clinical observations have established dichotomous roles for IL-17. For example, while IL-17 generally protects against certain fungal and bacterial infections, it can mediate pathology in allergic and autoimmune diseases [15, 16]. Case reports have associated deficient IL-17 responses with invasive aspergillosis [17, 18]. However, the contribution of IL-17 to host defenses against aspergillosis has not been well studied.

Originally described as a product of Th17 cells, subsequent studies established that other lymphoid subsets also produced IL-17, including CD8^+^ T cells, γδ T cells, natural Th17 cells, natural killer T cells and innate lymphoid cells [14]. Induction of lineage fate of IL-17-producing lymphoid cells requires the master transcription factor, RORγt (the gene product of *Rorc*), and *Rorc* knockout mice generally phenocopy IL-17-deficient mice [19]. However, RORγt also promotes survival of CD4^+^, CD8^+^ double positive thymocytes [20]. The heterodimeric cytokine, IL-23, promotes the maturation and expansion of Th17 cells and other IL-17-producing lymphocytes [21]. RORγt induces IL-23R and mice deficient in IL-23 generally have defects in the function of IL-17-producing lymphocytes [22, 23]. In addition, T cells capable of producing IL-17 independent of IL-23/IL-23R have been described [24].

Regarding myeloid-lineage cells, IL-17 production has been described in subsets of human and murine neutrophils [25]. Using murine models of acute and allergic pulmonary aspergillosis, we and others recently demonstrated that lung eosinophils elicited in response to challenge with live *A*. *fumigatus* or sensitization to *A*. *fumigatus* crude protein extracts (*Af* cpe) produce IL-17 [8, 9]. Eosinophils have also been implicated as a potential source of IL-17 within the airways of humans with asthma [26]. These findings may be particularly relevant because high levels of IL-17 have been correlated with symptom severity in allergic asthma [27], possibly due to promotion of neutrophilic airway inflammation [28]. In addition, in experimental models of asthma, mice lacking IL-17 signaling have decreased airway hyperresponsiveness and mucus production [29]. The goal of this study was to define the contributions of RORγt and IL-23 to eosinophil IL-17 production. We show that eosinophils express RORγt and IL-23R in acute and allergic models of pulmonary aspergillosis. However, using IL-23p19-deficient mice, we demonstrate that while *Aspergillus*-induced eosinophils express IL-23R, IL-23 is not required for eosinophil RORγt expression and IL-17 production.

## Results

### IL-17 expression by lung eosinophils in a modified mouse model of allergic pulmonary aspergillosis

**Fig 1:**
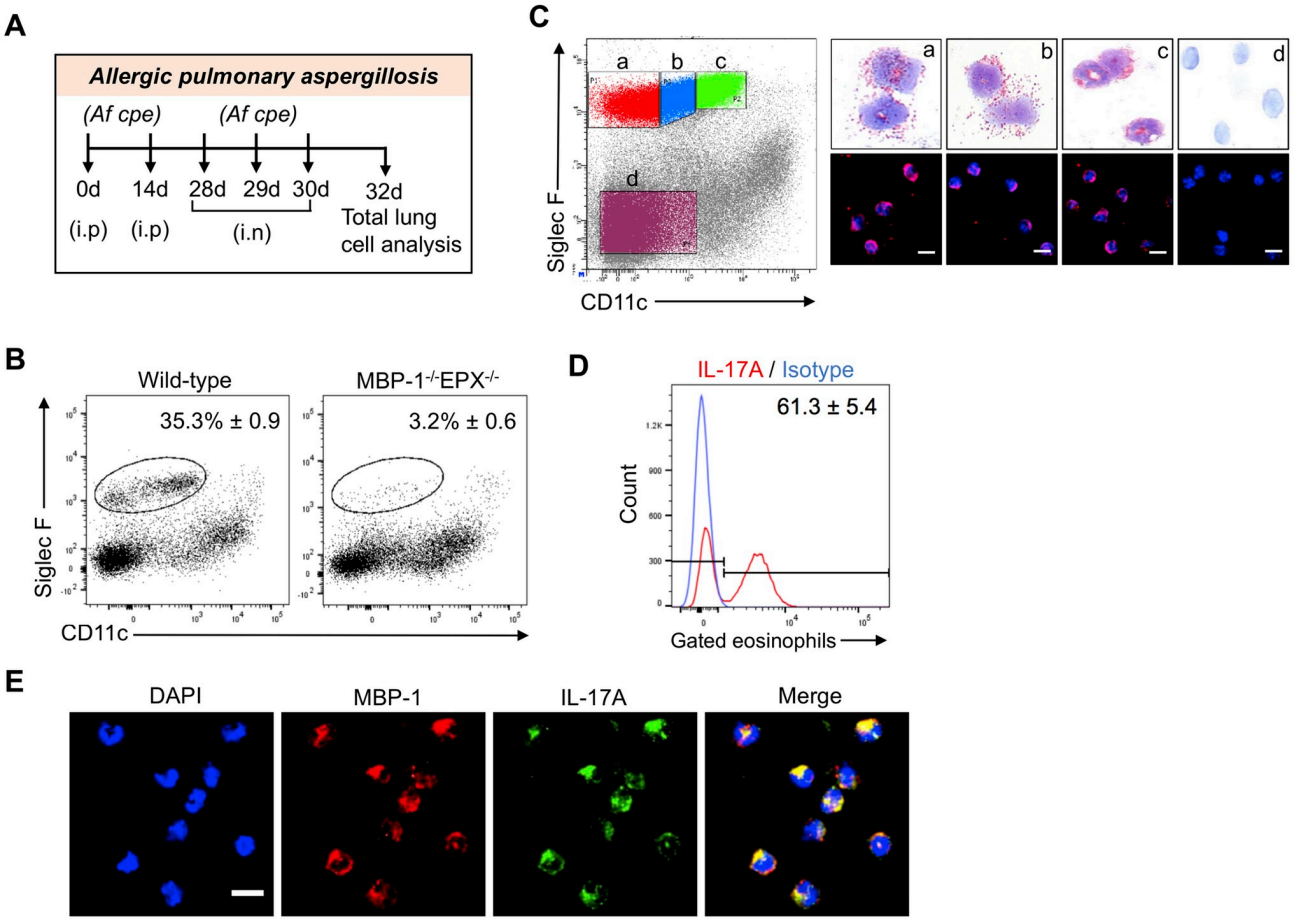
Production of IL-17 by pulmonary eosinophils in mice with allergic aspergillosis. (A) *Schematic of allergic pulmonary aspergillosis model*. Mice were sensitized intraperitoneally (i.p.) with 250 μg of *A*. *fumigatus* crude protein extract (*Af cpe*) on days 0 and 14. Two weeks later, mice received 3 daily intranasal (i.n.) challenges with 25μg of *Af cpe*. Two days after the 3rd challenge, the mice were euthanized, single cell lung suspensions were prepared, and analyzed for eosinophils and cytokines by flow cytometry with intracellular staining (ICS). (B) *Lung eosinophil recruitment*. Allergic aspergillosis was established in wild-type C57BL/6 and MBP-1^-/-^EPX^-/-^ and single cell lung suspensions were made as in Fig 1A. Lung eosinophils were analyzed by using cell surface markers (Siglec F/CD11c) after gating for live, CD45^+^ cells. Plot is representative of 2 independent experiments, each containing 5 mice. (C) *Sorted eosinophil subpopulations*. As in Fig 1B except subpopulations of Siglec F^+^ lung cells were gated and sorted according to level of expression of CD11c. As a control, a subpopulation of Siglec F^-^ cells was also sorted. Sorted cells were analyzed by light microscopy after Diff-Quik staining, and by immunofluorescence after staining for the eosinophilic cytoplasmic granule marker, MBP and the nuclear marker, DAPI. Representative of 2 independent experiments, each containing 5 mice. (D) *Intracellular cytokine staining (ICS)*. As in Fig 1B, except mice were treated with monensin intraperitoneally at 48 hours post-challenge to block cytokine secretion. Six hours following monensin treatment, mice were euthanized and lung single cells were prepared. Eosinophil IL-17 production was determined by ICS. Histograms show the shift in signal intensity for each cytokine compared to isotype control. FACS plots are representative of 3 experiments, each with 4–5 mice per group. The means ± SE of IL-17-staining cells combining each mouse experiment are shown next to the histograms. (E) *Confocal microscopy*. Allergic pulmonary aspergillosis was modeled in C57BL/6 mice as in Fig 1A. Two days following the 3^rd^ intranasal challenge, mice were euthanized and single cell lung suspensions were made. After staining for Siglec F and CD11c, the eosinophil population was enriched by sorting on a FACS. Cells were then permeabilized and stained intracellularly with anti-IL-17A (green), anti-MBP (red) and DAPI (blue). Merge: DAPI + MBP + IL-17A. Scale bar = 5μm. Images are representative of two independent experiments, each with three mice. Greater than 100 cells were examined in each experiment.

In our published allergic pulmonary aspergillosis protocol, eosinophils recruited to the lungs of sensitized C57BL/6 mice challenged with aerosolized *A*. *fumigatus* crude protein extract (*Af cpe*) produced IL-17 and IL-23 [8]. That protocol required a specialized aerosol chamber and large amounts of allergen. To circumvent these drawbacks, we examined the effect of pulmonary challenge via intranasal instillation of *Af* cpe on lung eosinophil recruitment and phenotype. Mice on the C57BL/6 background were sensitized with two biweekly intraperitoneal doses of 250 μg *Af* cpe and received three daily intranasal challenges with 25μg *Af* cpe (Fig 1A). Flow cytometric analysis of single cell preparations of the lungs of mice analyzed two days following the last challenge revealed the mice developed a robust eosinophilia (Fig 1B and S1 Fig). The gate used to define eosinophils was almost completely vacant when lung cells from eosinophil-deficient MBP-1^-/-^EPX^-/-^ mice were analyzed.

While high expression of CD11c on Siglec F^+^ cells is characteristic of alveolar macrophages [30], eosinophil populations expressing low to intermediate CD11c levels can emerge during allergic inflammation [31]. To confirm that these cells were eosinophils in our allergic pulmonary aspergillosis model, Siglec F^+^ lung cells were sorted based on level of expression of CD11c into three sub-populations: (a) CD11c ^**-**^, (b) CD11c ^low^, and (c) CD11c ^intermediate^ (Fig 1C). The sorted cells were then subjected to modified Wright Giemsa (Diff-Quik) and immunofluorescence staining for the eosinophilic granule protein, major basic protein (MBP). Cells from all three gates were predominantly eosinophils as determined by their bilobed and donut shaped nuclear morphology, presence of eosinophilic cytoplasmic granules and immunofluorescence staining for MBP. The negative control, cells from the Siglec F^-^ /CD11c^-^ gate, did not contain eosinophils as assessed by both Diff-Quik and immunofluorescence staining. Next, eosinophil expression of intracellular IL-17 was determined in this model of allergic pulmonary aspergillosis. As with our previous model [8], a high percentage of eosinophils stained positive for IL-17 by flow cytometry (Fig 1D) and confocal microscopy (Fig 1E).

### RORγt expression in lung eosinophils from mice with allergic and acute pulmonary aspergillosis

**Fig 2:**
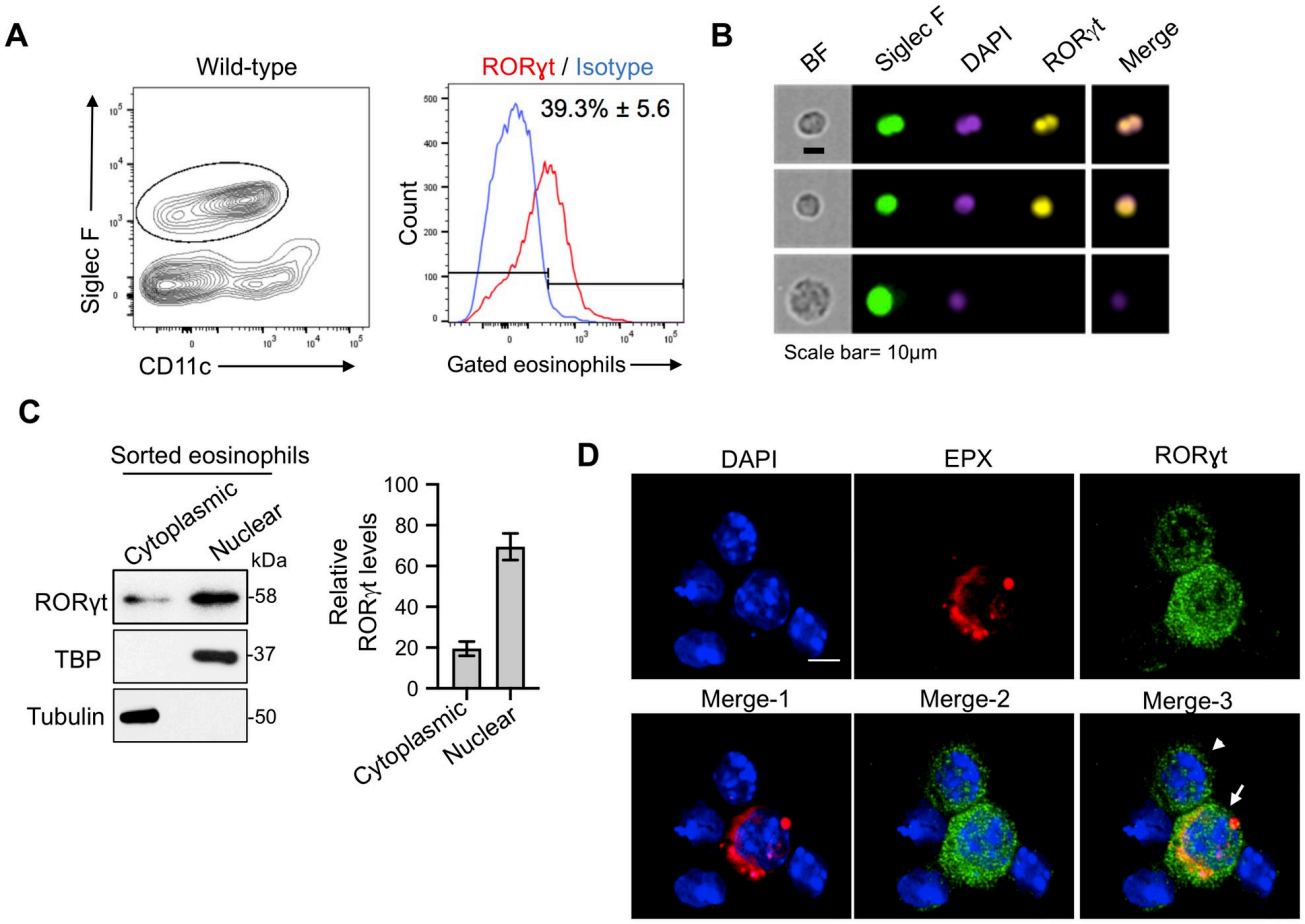
RORγt is expressed in lung eosinophils from mice with acute and allergic aspergillosis. (A) *Flow cytometry (allergy model)*. Allergic pulmonary aspergillosis was modeled as in Fig 1A. Lung cells were fixed, permeabilized and stained with anti-RORγt or isotype control. FACS plots are representative of 2 experiments, each with 4–5 mice per group. The means ± SE of RORγt-staining eosinophils combining each mouse experiment are shown next to the histogram. (B) *Imaging flow cytometry (allergy model)*. As is Fig 2A, except lung cells were analyzed by imaging flow cytometry after staining for Siglec F, DAPI (nuclear stain) and RORγt. Shown are representative output images from >100 analyzed cells. BF; Bright field. Scale bar, 10μm. (C) *Immunoblotting (allergy model)*. As in Fig 2A except eosinophils were purified by flow-sorting. Cytoplasmic and nuclear extracts were prepared by cellular fractionation, resolved by SDS-PAGE and analyzed by immunoblotting with primary antibodies against RORγt, TATA-binding protein (TBP) and α-tubulin. TBP was used as nuclear and α-Tubulin as cytoplasmic controls, respectively. Left panel shows representative immunoblots. Right panel shows the means ± SD of the relative density from 2 immunoblots probed for RORγt. (D) *Confocal microscopy (acute infection model)*. C57BL/6 mice were infected with 5 x 10^7^ *A*. *fumigatus* conidia. Two days post-infection, cells were harvested by BAL, permeabilized, and stained for the eosinophil marker, EPX (red), RORγt (green) and DAPI (blue). Merge-1: DAPI + EPX; Merge-2: DAPI + RORγt; Merge-3: DAPI + EPX + RORγt. Scale bar = 5μm. The arrow points to an eosinophil while the arrowhead points to a presumed neutrophil. Both cells exhibit RORγt staining.

To explore the mechanisms underpinning lung eosinophil IL-17 production, we investigated the contribution of RORγt. This transcription factor induces *IL-17* gene expression in T_H_17, T_C_17, γδ T cells, innate lymphoid cells, and neutrophils [25]. Using the allergic pulmonary aspergillosis model described above, nearly 40% of the lung eosinophils stained positive for RORγt by intracellular flow cytometry (Fig 2A). RORγt was observed in eosinophils by imaging flow cytometry. The top two panels of Fig 2B show representative images of sorted eosinophils exhibiting RORγt staining which co-localizes with the nuclear stain, DAPI, on the merge image. The lower panel shows an apparent alveolar macrophage (AMφ) which, while Siglec F positive, can be distinguished from eosinophils based on its larger size and nuclear morphology. This cell stains negative for RORγt. Note that the antibody to RORγt may also react with RORγ. As monensin was not added in these experiments, we could not determine whether eosinophils which expressed RORγt also expressed IL-17.

We next sought to validate RORγt expression in eosinophils by an alternative technique and to further examine the intracellular localization of this transcription factor. Lung eosinophils from mice with allergic pulmonary aspergillosis were sorted by flow cytometry and separated into nuclear and cytoplasmic fractions. While RORγt was detected in both fractions by immunoblotting, it was significantly enriched in the nucleus (Fig 2C). The veracity of the fractionation procedure was confirmed by immunoblotting for the nuclear and cytoplasmic proteins, tata-binding protein (TBP) and tubulin, respectively. The specificity of the RORγt antibody was confirmed by immunoblotting cytoplasmic and nuclear lysates obtained from thymocytes of RORγt^-/-^ and wild-type mice. As expected, a 58 kDa band (corresponding to the molecular size of RORγt [19]) was observed in lysates from nuclei of wild-type but not RORγt^-/-^ thymocytes (S2 Fig).

To determine whether eosinophils obtained from acutely infected lungs also express RORγt, we used our previously described acute pulmonary aspergillosis model in which mice receive a pulmonary challenge with live *A*. *fumigatus* conidia and then bronchoalveolar lavage (BAL) is performed 48 hours post-infection [8]. BAL cells were immunostained for RORγt, the eosinophil marker, EPX, and the nuclear stain DAPI. By confocal microscopy, the presence of intranuclear and cytoplasmic RORγt was observed in lung eosinophils (Fig 2D). Similar results were obtained using another eosinophil marker, MBP (S3 Fig). When quantitated, most eosinophils positively stained for RORγt (S3 Fig).

### Expression of mCherry in eosinophils from RORγt-mCherry reporter mice

**Fig 3:**
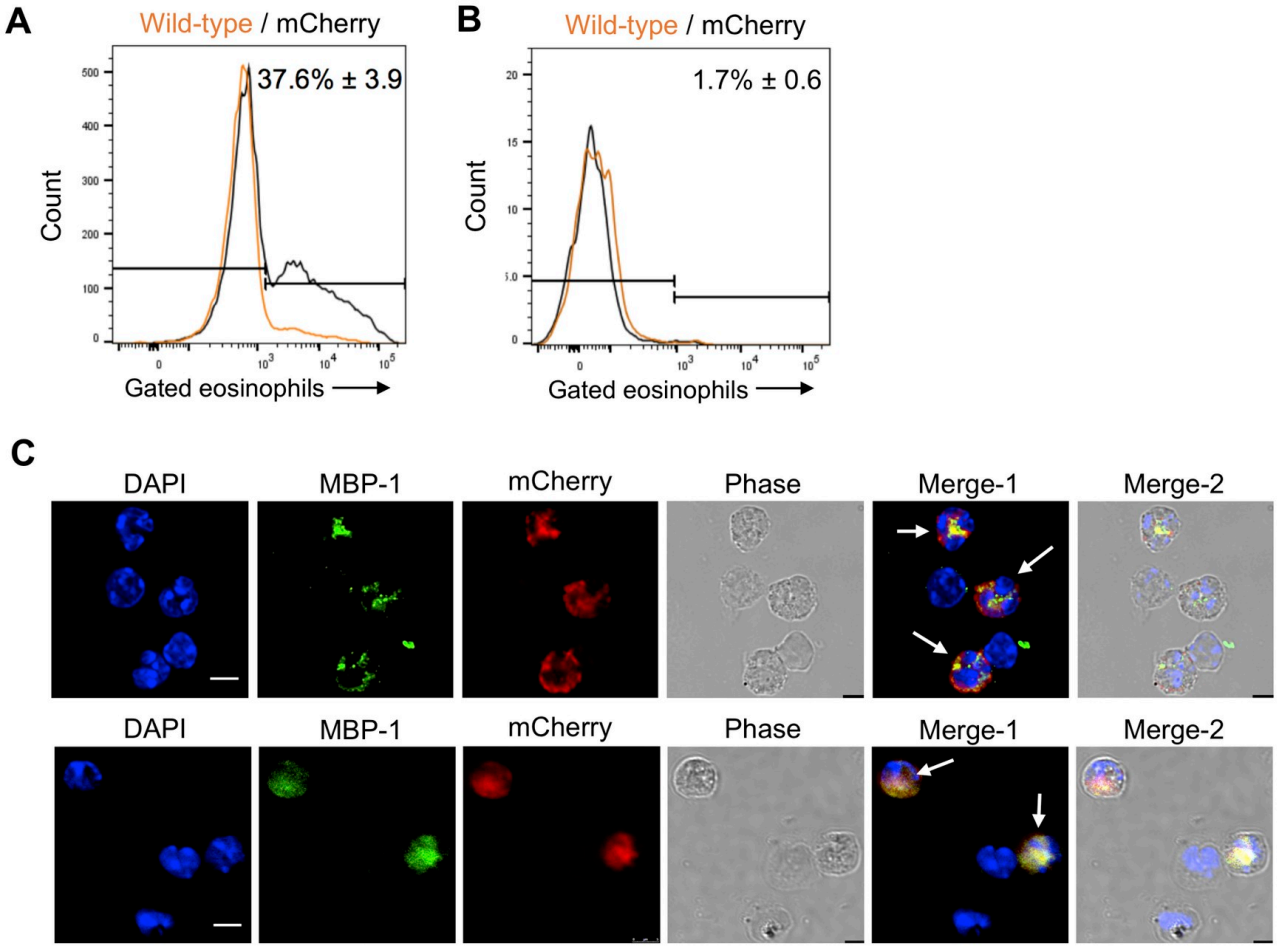
RORγt expression in lung eosinophils assessed using RORγt-mCherry reporter mice. (A) *Flow cytometry (allergy model)*. Allergic aspergillosis was induced in wild-type and RORγt-mCherry reporter mice as in Fig 1A. Eosinophils from lung cells were analyzed for expression of the mCherry signal. The plot demonstrates representative histograms of gated eosinophils from wild-type (orange) and RORγt-mCherry reporter (black) mice, respectively. The mean ± SE (n = 6 mice) of mCherry-positive eosinophils combining all the reporter mice experiments is shown next to the histogram. (B) *Flow cytometry (untreated mice)*. Lungs were harvested from untreated wild-type and mCherry mice and their eosinophils were analyzed for mCherry expression. FACS plots are representative of 2 experiments, each with 5–6 mice per group. The mean ± SE (n = 11 mice) of mCherry-positive eosinophils combining all the reporter mice experiments is shown next to the histogram. (C) *Confocal microscopy*. Upper Panel: BAL cells were harvested from RORγt-mCherry reporter mice with allergic aspergillosis induced as in Fig 1A. Cells were permeabilized and stained for the eosinophil marker, MBP (green) and the nuclear marker, DAPI (blue). The mCherry reporter is in red. Merge-1: DAPI + MBP + mCherry, Merge-2: DAPI + MBP + mCherry + Phase. Scale bar = 5μm.Lower Panel: RORγt-mCherry reporter mice were infected with 5 x 10^7^ *A*. *fumigatus* conidia. Two days post infection, cells were harvested by BAL and permeabilized and stained as in Fig 3B. Merge-1: DAPI + MBP + mCherry, Merge-2: DAPI + MBP + mCherry + Phase. Scale bar = 5μm.

As an independent means of assessing whether lung eosinophils express RORγt in our aspergillosis models, we used bacterial artificial chromosome (BAC) transgenic reporter mice which express mCherry under control of the *Rorc(t)* promoter. Cells which express the mCherry signal are predicted to also express RORγt. We sensitized and challenged the mCherry RORγt reporter mice with *Af* cpe and analyzed the lung eosinophils for the presence of mCherry signal by flow cytometry. Nearly 40% of the lung eosinophils expressed the mCherry reporter (Fig 3A). In contrast, mCherry expression was not seen in lung eosinophils obtained from naïve mice (Fig 3B). mCherry expression was also seen in BAL eosinophils examined by confocal microscopy in allergic and acute (Fig 3C) pulmonary aspergillosis models. For these experiments, eosinophils were identified based on positive immunostaining for the cytoplasmic marker MBP.

### Expression of IL-23R by lung eosinophils obtained from mice with allergic and acute pulmonary aspergillosis

**Fig 4:**
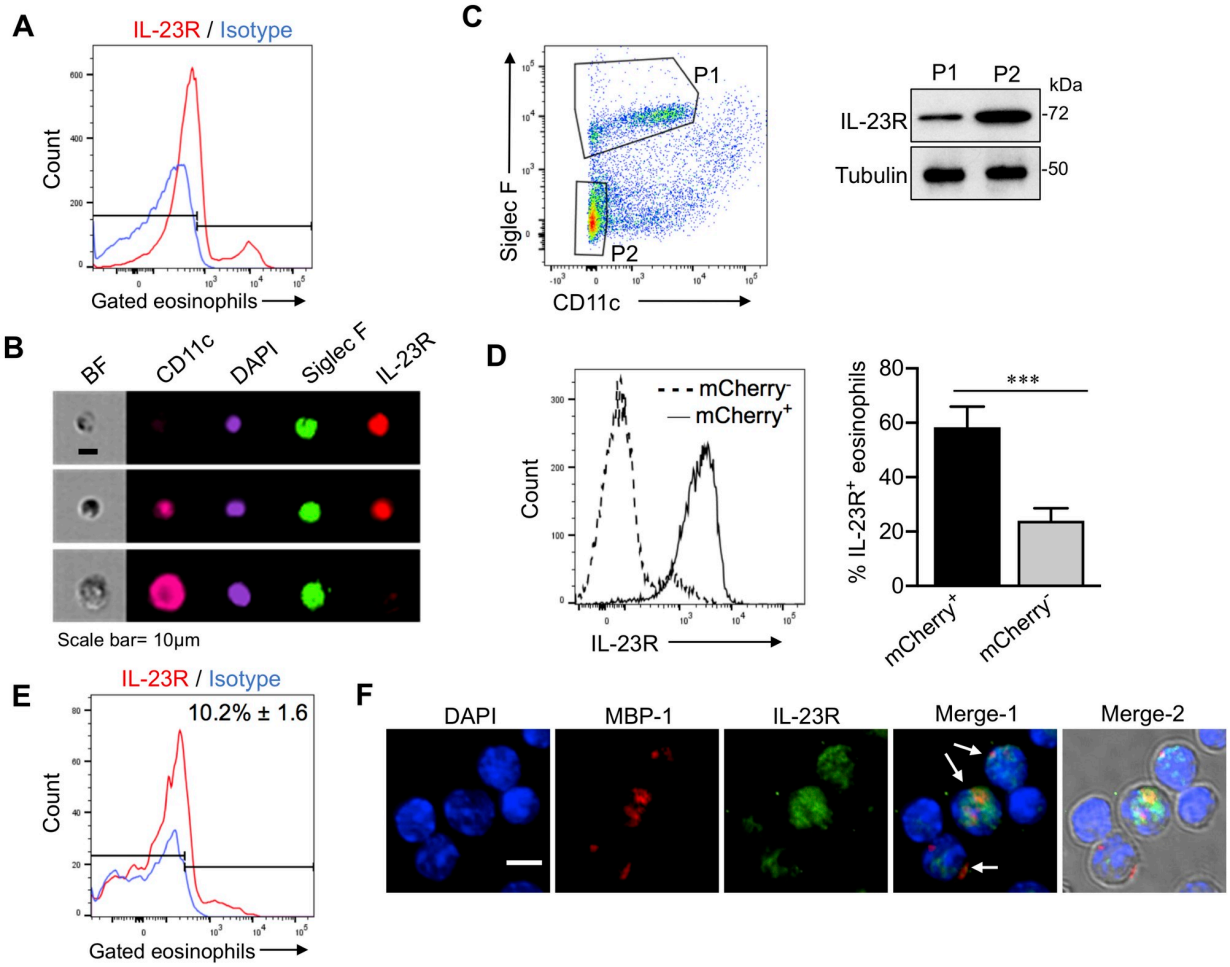
Eosinophil IL-23R expression from mice with acute and allergic pulmonary aspergillosis. (A) *Flow cytometry (allergy model)*. Allergic aspergillosis was induced in C57BL/6 mice as shown in Fig 1A. Single cells were prepared from harvested lungs and stained for CD45, CD11c, Siglec F and IL-23R. Live cells in the eosinophil gate were then analyzed by flow cytometry. A representative histogram is shown comparing IL-23R expression on eosinophils with an isotype control. The means ± SE (n = 5 mice) of IL-23R-positive eosinophils are shown next to the histogram. (B) *Imaging flow cytometry (allergy model)*. As in Fig 4A except cells were analyzed by imagining flow cytometry. Shown are representative output images (original magnification 20X). The top two panels demonstrate representative IL-23R^+^ eosinophils, one of which is CD11c^-^ and the other CD11c^int^. The lower panel shows a presumed alveolar macrophage (AMφ), distinguished from an eosinophil based on its larger size, high levels of CD11c expression and nuclear morphology. BF; Bright field. Cells shown are representative of >100 analyzed cells. Scale bar = 10μm. *(C) Immunoblotting (allergy model)*. Allergic aspergillosis was induced in C57BL/6 mice as in Fig 1A, following which lungs were harvested and single cell preparations were made. The left figure shows the sorting gates of the two different cell populations used for immunoblotting of IL-23R. P1: eosinophils, P2: SigF^-^CD11c^-^ cells. Whole cell extracts were made, resolved by SDS-Page and immunoblotted with primary antibodies for IL-23R and α-tubulin. The immunoblot is representative of 2 independent experiments. Tubulin was used as a loading control. (D) *Flow cytometry (allergy model*, *mCherry reporter mice)*. Allergic aspergillosis was induced in RORγt-mCherry reporter mice as described in Fig 1A. After harvesting lungs, single cell lung preparations were made. By flow cytometry, mCherry negative (mCherry^-^) and mCherry positive (mCherry^+^) lung eosinophils were analyzed for the presence of IL-23R. Left panel: Representative histograms of IL-23R expression in the mCherry^-^ and mCherry^+^ eosinophils. Right panel: Bar graphs showing the mean ± SEM percent IL-23R^+^ eosinophils in mCherry^+^ and mCherry^-^ eosinophils from two independent experiments, each of which had 4–5 mice (p <0.001 comparing the two groups). (E) *Flow cytometry (acute infection model)*. C57/BL6 mice were infected with 5x10^7^*Af293* conidia. Mice were euthanized 48 hours post-infection, single cell suspensions were made from harvested lungs, and analyzed for IL-23R expression as in Fig 4A. Histograms show the shift in IL-23R signal intensity in gated eosinophils. A representative histogram is shown comparing IL-23R expression on eosinophils with an isotype control. The means ± SE (n = 5–6 mice) of IL-23R-positive eosinophils are shown next to the histogram. (F) Confocal microscopy *(acute infection model)*. As in Fig 4D except cells were analyzed by confocal microscopy. Merge-1: DAPI + MBP (red) + IL-23R (green). Arrows point to eosinophils identified based on MBP staining. Merge-2: DAPI (blue) + MBP (red) + IL-23R (green) + brightfield. Scale bar = 10 μm.

The heterodimeric cytokine IL-23, acting through the IL-23R, contributes to and enhances the function of IL-17-producing lymphoid cells[14]. In our models of acute and allergic aspergillosis, lung eosinophils express IL-17 [8]. Therefore, we examined whether IL-23R was expressed on the surface of lung eosinophils. With the allergic model of aspergillosis, approximately one third of the lung eosinophils stained positive for IL-23R by flow cytometry (Fig 4A). IL-23 positive eosinophils were also observed by imaging flow cytometry (Fig 4B). In contrast, alveolar macrophages, which were distinguished from eosinophils based on their larger size, nuclear morphology and higher CD11c staining, were negative for IL-23R staining (Fig 4B).

We next determined IL-23 receptor expression on eosinophils by immunoblotting (Fig 4C). Lung eosinophils were flow-sorted from mice with allergic aspergillosis as described in Methods. As a positive control, cells were sorted from the Siglec F^**-**^ CD11c^**-**^ gate); this gate includes cell types known to express IL-23R, including neutrophils, γδ T cells and T_h_17 cells [25, 32, 33]. We found IL-23R was expressed in eosinophils as well as in cells from SigF^-^CD11c^-^ gate (Fig 4C).

As with other Th17-specifying genes such as *il17a*, *il17f*, *il21 and il22*, *Rorc* also regulates *Il23r* gene expression[34]. We therefore sought to determine whether there was a correlation between RORγt and IL-23R expression in lung eosinophils. RORγt–mCherry reporter mice were sensitized and challenged with *Af* cpe following which, lung eosinophil IL-23R expression was determined by flow cytometry. As observed in our previous experiments (Fig 3A), mCherry-positive and mCherry-negative eosinophil populations were present (Fig 4D). Importantly, most of the mCherry-positive eosinophils also expressed IL-23R whereas the mCherry-negative eosinophils were largely IL-23R negative (Fig 4D). These results suggest that RORγt may drive IL-23R expression in eosinophils. We also examined the expression of IL-23R in lung eosinophils 2d post challenge with live *A*. *fumigatus* conidia. By flow cytometry, we found a distinct population of IL-23R^+^ lung eosinophils comprising approximately 10% of the gated eosinophils (Fig 4E). IL-23R was also detected on a subpopulation of the eosinophils by confocal microscopy (Fig 4F).

### IL-17 and IL-23r gene expression in lung eosinophils from mice with allergic aspergillosis

**Fig 5:**
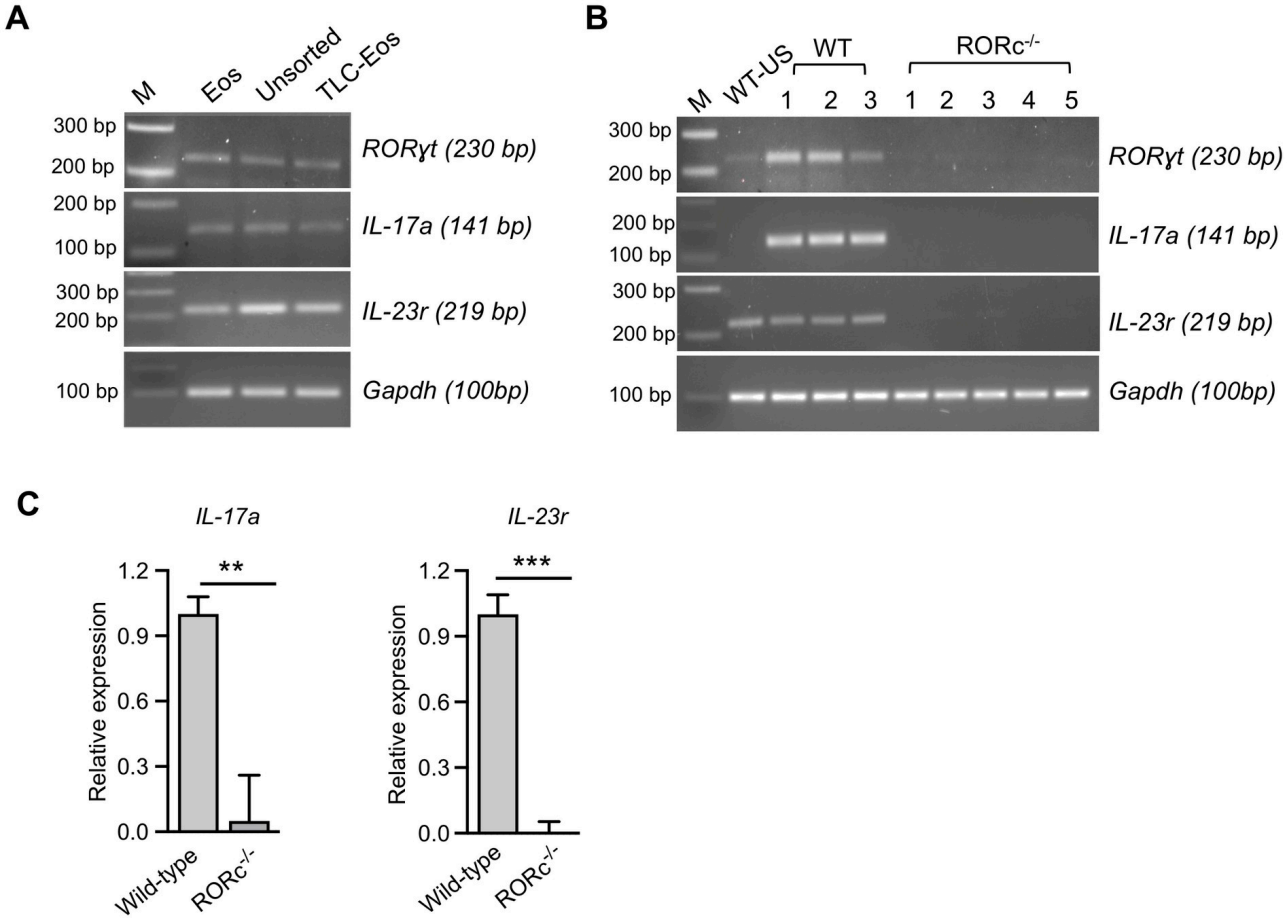
RORγt, IL-17 and IL-23r gene expression in lung eosinophils from wild-type and RORc^-/-^ mice with allergic aspergillosis. (A) Allergic aspergillosis was induced in C57BL/6 mice as in Fig 1A, following which lungs were harvested and single cell preparations were made. The total lung cells (TLC) were separated into two populations by flow-sorting: eosinophils (Eos) and the rest of the lung cells (TLC—Eos). Unsorted TLC from a lung of allergic mice was also collected. *RORγt*, *IL-17a*, *IL-23r* mRNA in the three cell populations was analyzed by RT-PCR. *Gapdh* served as a loading control. These results are representative of two independent experiments with n = 7–8 mice per experiment. The indicated DNA ladders served as base pair (bp) markers (M). (B) Allergic aspergillosis was induced in wild-type (WT, n = 3) and RORc^-/-^mice (n = 5), following which mRNA was isolated from harvested lungs and analyzed by RT-PCR for *RORγt*, *IL-17a*, *IL-23r* and *Gapdh*. DNA markers are as in Fig 5A. Wild-type unstimulated (WT-US) refers to results obtained using lung mRNA from a naïve (non-allergic) mouse. The remaining lanes show results from mRNA isolated from the lungs of individual WT and RORc^-/-^ mice with allergic aspergillosis. (C) Real time quantitative RT-PCR for *IL-17a and IL-23r* was performed using mRNA isolated from lung cells obtained from wild-type and RORc^-/-^mice with allergic aspergillosis. Results are expressed as means + SEM (n = 4 mice/group) of relative gene expression compared with the internal control, *Gapdh*. ** p = 0.006; *** p = 0.*001)*.

To determine whether protein expression was accompanied by active gene expression, we sorted eosinophils from the lungs of wild-type mice with allergic aspergillosis and analyzed *RORγt*, *IL-17*, and *IL-23r* mRNA by reverse transcriptase (RT)-PCR. We found robust expression of all three genes in the sorted eosinophils. As expected, based on literature demonstrating that in lungs stimulated by *A*. *fumigatus*, eosinophils are not exclusive producers of IL-17[8, 9, 25], total lung cells (unsorted) and lung cells in the eosinophil-negative gate also expressed the above-mentioned genes (Fig 5A*)*.

Next, we sensitized and challenged wild-type and RORc^-/-^ mice with *Af* cpe and analyzed total lung cells for RORγt, *IL-17a*, and *IL-23r* expression by RT-PCR. As RORγt is present in CD4^+^CD8^+^ double positive thymocytes [20], we first confirmed the phenotype of RORc^-/-^ mice by analyzing *RORγt* expression in thymocytes of wild-type and RORc^-/-^ mice by RT-PCR. As expected, there were undetectable *RORγt* transcripts in the knockout mice (S4 Fig). *RORγt*, *IL-17a*, and *IL-23r* expression was not detectable in total lung cells from RORc^-/-^ mice compared to its wild-type counterpart (Fig 5B). These data were confirmed by quantitative real-time RT-PCR (Fig 5C).

### Role of IL-23p19 in inducing expression of RORγt and IL-17 in eosinophils

**Fig 6:**
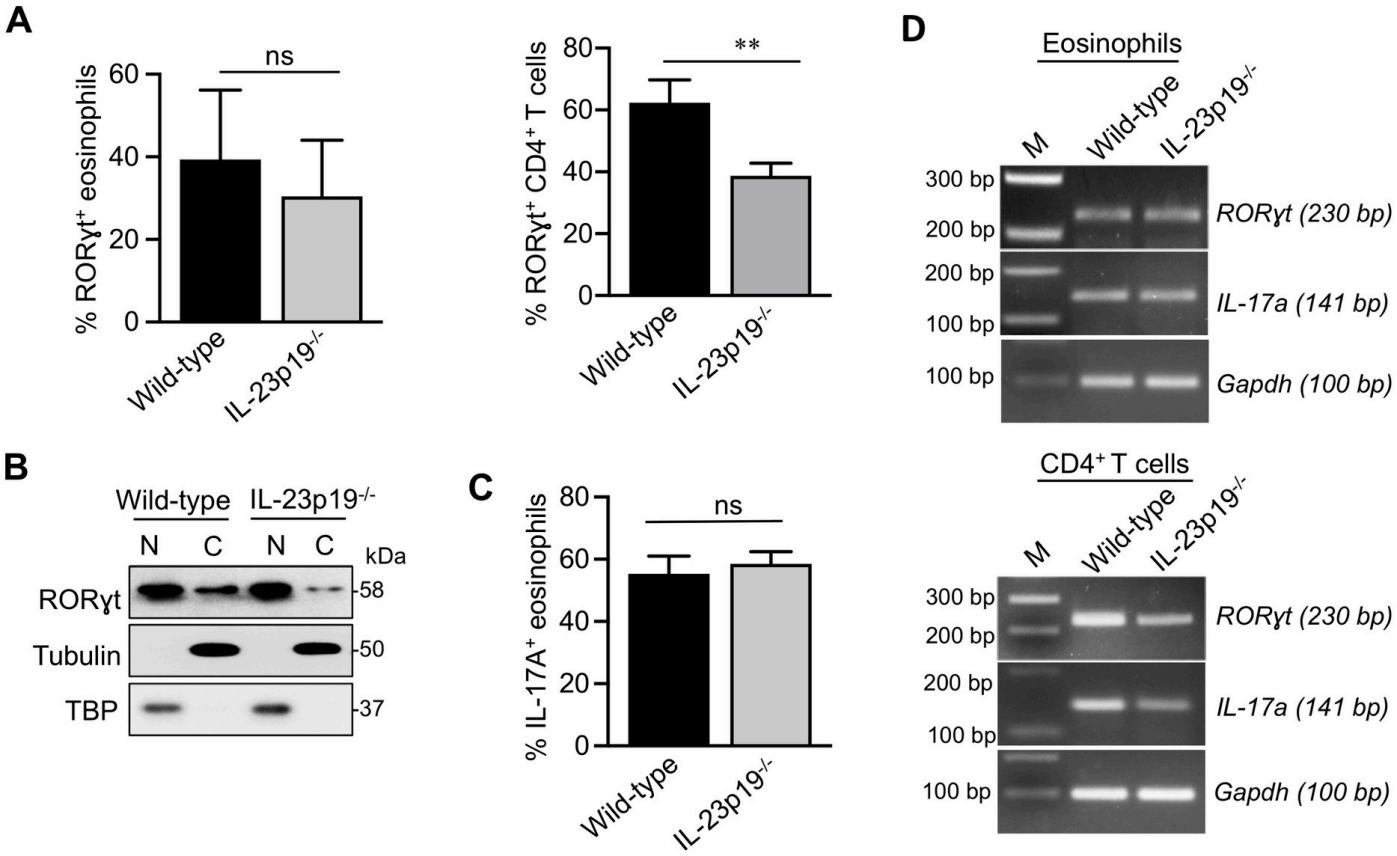
RORγt and IL-17 expression in lung eosinophils and CD4^+^ T cells from wild-type versus IL-23p19 deficient mice with allergic aspergillosis. (A) *RORγt expression by flow cytometry*. Allergic aspergillosis was induced in wild-type and IL-23p19^-/-^ mice as described in Fig 1A. Upper panel: Gated lung eosinophils were analyzed for RORγt staining by flow cytometry with ICS. The bar graphs show the percent RORγt^+^ eosinophils in wild-type and IL-23p19^-/-^ mice in three separate experiments, each of which had 4–5 mice per group. Data are means ± SEM (ns, not significant). Lower panel: As in the upper panel except lung CD4^+^ T cells, gated based on positive staining for CD45, CD3 and CD4, were stained for RORγt. Data shown in the bar graphs are means ± SEM of an experiment with 4 mice/group (p <0.01). (B) *Immunoblotting for ROR*γ*t*. Allergic aspergillosis was induced in wild-type and IL-23p19^-/-^ mice as shown in Fig 1A. Eosinophils were purified from total lung cells by FACS-sorting, separated into cytoplasmic (C) and nuclear (N) fractions and analyzed for RORγt by immunoblotting, as described in Fig 2C. TBP and α-Tubulin served as nuclear and cytoplasmic controls, respectively. (C) *IL-17 expression by flow cytometry*. Allergic aspergillosis was induced in wild-type and IL-23p19^-/-^ mice as shown in Fig 1A. Lungs were harvested, and single cell preparations were made. Gated lung eosinophils were analyzed for IL-17 staining by flow cytometry with ICS. The bar graphs show the percent eosinophils in wild-type and IL-23p19^-/-^ mice in two separate experiments, each of which had 4–5 mice per group. Data are means ± SEM (ns, not significant). (D) *RT-PCR analysis*. Allergic aspergillosis was induced in wild-type and IL-23p19^-/-^ mice as shown in Fig 1A. Lung eosinophils (left panel) and CD4^+^T cells (right panel) were purified by flow-sorting following which their RNA was analyzed by RT-PCR for the expression of *RORγt* and *IL-17a*. *Gapdh* expression served as a loading control. M: DNA markers, as in Fig 5A.

Along with IL-6 and TGF-β, IL-23 participates in activating Jak2-Stat3 signaling pathways to upregulate the expression of RORγt in lymphoid cells[35]. We investigated the contribution of IL-23 to eosinophils RORγt expression and IL-17 production by using IL-23p19^-/-^ mice [22]. In the allergic pulmonary aspergillosis model, we found similar expression of RORγt when comparing lung eosinophils from IL-23p19^-/-^ and wild-type mice by flow cytometry (Fig 6A). However, as expected [34], RORγt expression was greatly reduced in CD3^+^CD4^+^ T cells from IL-23p19^-/-^ mice compared to the wild-type mice (Fig 6A and S5 Fig). The percentage and total number of eosinophils in the lungs were similar comparing IL-23p19^-/-^ and wild-type mice (S6 Fig).

To further validate eosinophil RORγt expression did not require IL-23 signaling, we induced allergic aspergillosis in IL-23p19^-/-^ and wild-type mice. Lung eosinophils were then purified by FACS, subjected to cellular fractionation and immunoblotted for RORγt. We found similar nuclear expression of RORγt comparing eosinophils from wild-type and IL-23p19^-/-^ mice (Fig 6B). By flow cytometry, similar IL-17A production was found in eosinophils from wild type and IL-23p19^-/-^ mice in the allergic pulmonary aspergillosis model (Fig 6C). Finally, by RT-PCR, we determined *RORγt* and *IL-17a* gene expression (Fig 6D) in FACS- sorted lung eosinophils and CD4^+^ T cells from wild-type and IL-23p19^-/-^ mice with allergic aspergillosis. We found transcript levels of *RORγt* and *IL-17a* were similar in eosinophils from wild-type and IL-23p19^-/-^ mice. In CD4^+^ T cells, we observed that both *RORγt* and *IL-17a* gene expression was reduced in IL-23p19^-/-^ mice compared to that of wild-type (Fig 6D). Our observations in CD4^+^T cells are in line with previously published reports where authors have shown the reduced expression of *RORγt* and *IL-17a* mRNA in CD4^+^ T cells from IL-23p19^-/-^ mice [36, 37]. Together these data suggest that lung eosinophils express RORγt and IL-17a in mice with allergic aspergillosis. While RORγt is required for the production of IL-17a, IL-23 is dispensable for lung eosinophils to express RORγt and IL-17a.

## Discussion

In acute and allergic pulmonary aspergillosis models, we and others have shown that lung eosinophils elicited in response to challenge with live *A*. *fumigatus* and sensitization to *A*. *fumigatus* crude protein extracts produce IL-17 [8, 9]. Moreover, eosinopenic mice with acute aspergillosis had significantly reduced levels of IL-23 heterodimer in their bronchoalveolar lavage fluid compared to their wild-type counterparts [8]. These findings suggest eosinophils are key drivers of the IL-23/IL-17 axis during pulmonary aspergillosis. Here, we studied intrinsic factors that promote eosinophil IL-17 production in acute and allergic aspergillosis. We examined the mechanistic basis for eosinophil IL-17 production in pulmonary aspergillosis, focusing on the roles of RORγt, IL-23 and IL-23R.

Strong evidence implicates RORγt as the master transcription factor indispensable for inducing IL-17-producing lymphoid cells [19, 38]. In addition, under certain conditions, neutrophils make RORγt and IL-17 [25]. Using multiple approaches including flow cytometry with intracellular staining, imaging flow-cytometry, immunoblotting and confocal microscopy, we identified a subpopulation of lung eosinophils from mice with allergic and acute aspergillosis that express RORγt. We could not use RORγt-GFP reporter mice to further demonstrate eosinophil RORγt expression because eosinophils have autofluorescence which overlaps with GFP [39]. However, we were able to take advantage of newly created RORγt-mCherry reporter mice to confirm eosinophil expression of RORγt. In Th17 and other IL-17-producing lymphoid cells, expression of RORγt is limited to the nuclear compartment, but in neutrophils, RORγt is found in both nuclear and cytoplasmic compartments [25]. Moreover, stimulation of neutrophils with IL-6 and IL-23 results in translocation of cytoplasmic RORγt to the nucleus[25]. Eosinophils also exhibit both nuclear and cytoplasmic RORγt, as evidenced by cell fractionation studies and confocal microscopy. However, most of the eosinophil RORγt resides in the nucleus. Future studies will be needed to determine the factors activating RORγt nuclear translocation and subsequent IL-17 production in eosinophils.

The intrinsic ability of myeloid cells to produce IL-17 cytokines has been controversial. IL-17 gene expression and protein production has been observed in mouse and human neutrophils by some investigators [25, 33]. In contrast, Tamassia et al., failed to detect IL-17 in human neutrophils from healthy donors and active psoriatic patients [40]. Moreover, neutrophils stimulated with IL-6 plus IL-23 were devoid of transcriptionally active histone marks at the *IL-17a* and *IL-17f* loci which are present in Th17 cells [40]. Furthermore, a population of human primary mast cells that contain IL-17 but do not express RORγt was described; these cells capture extracellular IL-17A secreted by other cells and then store the cytokine in their intracellular granules [41]. However, the data presented in this manuscript and our previous publication [8] strongly suggest that lung eosinophils synthesize IL-17 following stimulation with *A*. *fumigatus* and antigens derived from the fungus. Multiple complementary techniques were used to detect RORγt and IL-17, including the use of IL-17 and RORγt reporter mice. Finally, the generalizability of the findings is suggested by the similar results seen using distinct acute and allergic models of aspergillosis.

In Th17 cells, the heterodimeric cytokine IL-23 acts through the IL-23R to drive and sustain IL-17 expression [32]. IL-23 also promotes survival of myeloid cells at sites of infection by a mechanism independent of IL-17 [42]. RORγt is known to positively regulate IL-23R gene expression [23, 43]. We observed IL-23R expression on eosinophils too in our models of acute and allergic aspergillosis. Moreover, in experiments with RORγt-mCherry reporter mice with allergic aspergillosis, we found a significant correlation between mCherry and IL-23R expression in lung eosinophils. Despite the induction of IL-23R, no significant differences were observed comparing allergic lung eosinophils from wild-type and IL-23p19^-/-^ mice with regards to eosinophil recruitment, RORγt expression and IL-17 production. If these data can be extended to humans, they suggest that blocking antibodies to IL-23 and IL-23R would not be effective at reducing eosinophil IL-17 production in allergic lung disease.

The finding of IL-23-independent IL-17 production in eosinophils has also been described in other cell types. A population of γδT cells expressing both RORγt and IL-23R yet can produce IL-17 independent of IL-23/IL-23R signaling was observed in colonic lamina propria [24]. Moreover, in a murine model of laser-induced experimental choroidal neovascularization, IL-23-independent IL-17 production by γδT cells and innate lymphoid cells was demonstrated [44]. Finally, production of IL-17 independent of IL-23R gene expression was reported in human enthesis γδT cells obtained during surgery [45].

The mechanisms by which RORγt is induced in eosinophils and the anatomical location where transcription is turned on remain to be determined. RORγt does not appear to be constitutively expressed in eosinophils as in the RORγt-mCherry reporter mice, unstimulated lung eosinophils were negative for mCherry expression. Interestingly, endogenous RORγt ligands which can act as non-canonical activators have been described, including oxysterols and bile acid metabolites [46, 47]. Just as there are subsets of lymphocytes, such as Th17 and Tc17 cells, in which RORγt and subsequent IL-17 expression is turned on, we propose there is an IL-17-producing “Eo_17_” subset of eosinophils that is induced under specific conditions in the host. More data are needed though before this term can be added to the immunology lexicon.

Regardless of the mechanism of activation, our data suggest that RORγt could serve as a drug target in persons with allergic aspergillosis. Identification of the endogenous agonist ligand(s) of RORγt in allergic eosinophils and determining how these ligands activate RORγt may provide insights into the design and development of RORγt modulators that block IL-17 production in eosinophils and inhibit pathology in allergic pulmonary aspergillosis. Indeed, antagonists of RORγt which suppresses IL-17- and IL-23-mediated inflammation are under preclinical investigation for the treatment of autoimmune and other inflammatory diseases [43, 48]. Finally, while RORγt is recognized as the master regulator of Th17 differentiation, it does so in concert with other transcription factors, including STAT3, IRF4, BATF, and RUNX1 [49, 50]. The contribution of these factors to transcription regulation of *Il17a* and *Il17f* in eosinophils requires study.

Asthma is a complex and heterogenous disease [51]. Clinical and experimental data demonstrate that eosinophils contribute to the airway hyperreactivity seen in asthmatic individuals that have allergic airway disease [8, 10, 11, 51]. Moreover, bronchoalveolar fluid IL-17 levels are increased in severe asthma [52], and there appears to be a subset of patients for whom IL-17 cytokines play pathogenic role [51]. However, in addition to eosinophils, numerous other IL-17-producing cell types are found in the lungs. Thus, a key question is whether IL-17 specifically derived from eosinophils plays a significant pathological role. Addressing this knowledge gap should help inform whether approaches targeting eosinophil production of RORγt and IL-17 are likely to be successful.

Allergic airway disease is believed to result from abnormal immune responses stimulated by environmental antigens. Previously, we used a model of allergic pulmonary aspergillosis that required a specialized aerosol chamber and relatively large amounts of antigen for the three pulmonary challenges [8]. In the present study, we simplified the method of inducing allergic pulmonary aspergillosis by changing the challenge to direct aspiration of intranasally administered antigens. The extent of lung eosinophilia observed using this easier and more economical allergic aspergillosis model was similar to previously described models from our lab and others [8, 53]. Importantly, we again observed expression of IL-17A by the majority of lung eosinophils elicited in the new allergic aspergillosis model. Moreover, consistent with previously published data with ova and house dust mite induced allergies [31, 54], the Siglec F^+^ eosinophils could be divided into subpopulations based on relative expression levels of CD11c. Other models of allergic aspergillosis have been described including one featuring repetitive intranasal instillation of live *A*. *fumigatus* conidia [55]. In that model, eosinophil-deficient mice had decreased expression of IL-17A.

In conclusion, we elucidated intrinsic molecular factors responsible for IL-17 production by eosinophils. We demonstrated that lung “Eo_17_” eosinophils express RORγt, the master transcription factor which drives IL-17 production in lymphoid cells. Challenge of lungs with live *A*. *fumigatus* or its antigens skews lung eosinophils towards the Eo_17_ phenotype by a non-canonical pathway independent of IL-23/IL-23R signaling. Elucidating the mechanisms by which Eo_17_ cells are stimulated could inform therapeutic approaches for treating allergic lung diseases in which eosinophilia is a major feature.

## Methods

### Ethics statement

Experimental procedures with mice were approved by the University of Massachusetts Medical School (protocol A1802) and the NYU School of Medicine (protocol IA15-01410) Institutional Animal Care and Use Committees in accordance with National Institutes of Health guidelines for the care and use of laboratory animals.

### Mice

All mice were on the C57BL/6 background and were bred and housed in specific-pathogen free conditions at the animal facilities in the University of Massachusetts Medical School. Mice of both genders were used and were generally six to ten weeks old at the start of the experiment. Wild-type C57BL/6J and RORγt^-/-^ mice [19] were purchased from The Jackson Laboratory (Bar Harbor, ME) and bred in house. Eosinophil-deficient MBP-1^-/-^EPX^-/-^ mice were obtained from James Lee and Elizabeth Jacobson (Mayo Clinic, Phoenix, AZ [56, 57]. IL-23p19^-/-^ mice were a gift from Nico Ghilardi (Genentech, South San Francisco, CA) [22]. The RORγt-mCherry reporter strain was generated by cloning mCherry-IRES-CreERT2-pA in frame with the initiation codon of *Rorc(t)*, which encodes the RORγt isoform of the nuclear receptor. Mouse founder lines were screened for mCherry reporter activity of *in vitro* polarized Th17 cells (positive control) compared to Th2 cells (negative control) derived from naïve CD4^+^ T cells sort-purified from blood samples. Wild-type and control mice were bred and housed in the same animal room.

### Murine allergic and acute aspergillosis models

The murine allergic aspergillosis model was as in previous studies [8], except the pulmonary challenges with *Aspergillus fumigatus* crude protein extract (*Af cpe*, Greer Laboratories, Lenoir, NC) were via the intranasal (IN) rather than aerosolized route. Briefly, mice were sensitized with 250μg of *Af* cpe administered via intraperitoneal (IP) injections on days 0 and 14. Mice were lightly anesthetized with isoflurane (Patterson Veterinary) and challenged IN with 25μg of *Af* cpe on days 28, 29, and 30. On day 32, mice were euthanized, and their lungs were harvested (Fig 1A).

The murine acute pulmonary aspergillosis model was also as described [8, 58, 59]. Briefly, conidia from *A*. *fumigatus* strain *Af*293 (Fungal Genetics Stock Center, Manhattan, KS) were harvested after growth on Sabouraud dextrose agar (Remel, San Diego, CA) slants and purified by two rounds of filtration through a 40μm nylon mesh. Conidial suspensions were washed three times in PBS containing 0.01% Tween-20. Mice were anesthetized as above and infected orotracheally with 5x10^7^ conidia. Mice were euthanized two days post fungal challenge and their lungs were harvested. For experiments measuring intracellular cytokine expression, mice received an IP injection of 500 μg of monensin (Sigma-Aldrich) 48 hours post *Af cpe* or conidia challenge [8]. Six hours following monensin treatment, mice were euthanized, and their lungs were harvested. Monensin was not used in experiments examining RORγt staining.

### Flow cytometry and cell sorting

Lung single cell suspensions were prepared using the MACS lung dissociation kit (Miltenyi Biotec) [8]. Leukocytes were sequentially enriched on a Percoll (GE Healthcare) gradient, incubated with Fc block (2.4G2, BD Pharmingen) and stained with a viability dye (Fixable Viability Dye eFluor 780, eBioscience) for 30 minutes at 4°C. Cells were washed with FACS buffer (1% BSA/PBS), stained at 4°C for cell surface markers (Table 1), washed twice to remove unbound antibody conjugates and fixed in 4% paraformaldehyde (Electron Microscopy Sciences) for 20 minutes. For intracellular cytokine staining, cells were permeabilized using commercial kits (BD Perm/Wash and BD Cytofix/Cytoperm, BD Pharmingen) according to the manufacturer’s instructions, washed twice and incubated with labeled antibodies (Table 1). Similarly, for staining of nuclear markers, we used the eBioscience Foxp3/Transcription Factor Staining Buffer Set (Invitrogen). Cells were stained for RORγt (Table 1) according to the manufacturer’s instructions. Fluorescence minus one and/or isotype controls were included in all experiments. Flow cytometry data were acquired with an LSRII cytometer and analyzed using FlowJo v10 Software (Tree Star Inc.). At least 100,000 events were collected per sample.

**Table 1:** Antibodies used for flow cytometry.

Cell Marker-fluorochrome conjugate	Clone	Manufacturer	Isotype
CD45 PerCP-Cy5.5	30-F11	BD Biosciences	Rat IgG2b
Siglec F-BV510	E50-2440	BD Biosciences	Rat IgG2a
CD11c PE-Cy7	N418	BioLegend	Armenian Hamster IgG
CD3-FITC	17A2	BD Biosciences	Rat IgG2b, κ
CD4-PE	GK1.5	eBiosciences	Rat/IgG2b, κ
IL-23R-APC	12B2B64	BioLegend	Rat IgG2b, κ
IL-17A-AF700	TC11-18H10	BD Biosciences	Rat IgG1, κ
RORγt-BV421	Q31-378	BD Biosciences	Mouse IgG2a, κ

Cells were sorted on a Becton Dickinson FACS Aria II cell sorter using an 85μm nozzle operated at 45psi. For eosinophil sorting, lung cells were stained with surface markers CD45, Siglec F and CD11c along with the live dead stain (Fixable Viability Dye eFluor 780). Sorted eosinophils were singlet cells (based on FSC-H vs FSC-A), which were live, CD45^+^, Siglec F^+^, and CD11c^-/low/intermediate^ (S1 Fig). For CD4^+^ T cell sorting, we sorted cells which were singlets, live, CD45^+^, CD3^+^, and CD4^+^.

### Cytological staining

Cells were counted and cytospun onto poly-L-Lysine coated slides (Shandon Cytoslides, Thermo Scientific) at 800 rpm for 3 minutes (Shandon Cytospin 2). Eosinophils were then differentiated by cytological staining using a commercial kit (Diff-Quik, Polysciences, Warrington, PA) per the manufacturer’s instructions. Finally, slides were mounted with Entellan Mounting Medium (Electron Microscopy Sciences).

### Confocal microscopy

Confocal microscopy was performed as described [8], with slight modifications. Briefly, single cell lung suspensions were prepared as for flow cytometry. Cells were counted, fixed with 2% paraformaldehyde, washed thrice with PBS and cytospun onto slides. Cells were then permeabilized with 0.01% Triton-X/PBS, washed thrice and blocked with 10% donkey serum (1 hour, room temperature). Cells were sequentially stained with goat polyclonal anti-MBP in 5% donkey serum (4°C, 3 hours), and Alexa fluor 594 donkey anti- goat Fab2 (1 hour, room temperature). Next, cells were stained with rabbit polyclonal antibody IL-17A (4 hours, 4°C), and Alexa fluor 647-labeled donkey anti-rabbit fab2 fragment (45 min., room temperature). Antibodies used are listed in Table 2. The DNA stain 4′,6-diamidino-2-phenylindole (DAPI, Sigma) was used to stain cell nuclei. Slides were mounted with Pro-Long Gold mounting media (Life Technologies). Confocal images were acquired on a Leica SP8 Confocal microscope with a 63x oil immersion objective lens in sequential scan mode.

**Table 2:** Antibodies used for confocal microscopy.

Cell Marker	Fluorochrome conjugate	Manufacturer / Catalog no.
Goat polyclonal anti-MBP	None	Santa Cruz/109316
Goat polyclonal anti-EPX	None	Santa Cruz/19148
Goat polyclonal IgG	None	Santa Cruz/3887
Donkey anti-goat fab2	Alexa fluor 594	Abcam/150140
Rabbit polyclonal anti-IL-17A	None	Abcam/79065
Rabbit polyclonal anti-RORγt	None	Abcam/78007
Rabbit polyclonal anti-IL-23R	None	Abcam/175072
Rabbit polyclonal IgG	None	Abcam/27478
Donkey anti-rabbit fab2	Alexa fluor 647	Abcam/181347

### Imaging flow cytometry

Lung cells, obtained from mice with allergic aspergillosis, were stained with a live-dead stain (Fixable Viability Dye eFluor 780) and the cell surface markers CD45, Siglec F, CD11c, IL-23R. Where indicated, cells were permeabilized and intracellularly stained for RORγt. Nuclei were distinguished by DAPI-staining. Brightfield and fluorescent images of cells were obtained with an Amnis/Flowsight Imaging Cytometer at 20X magnification and analyzed using IDEAS software (Luminex).

### Western blot and immunoblotting

Lung eosinophils, obtained from C57BL/6 mice sensitized and challenged with *Af* cpe, were purified by fluorescence-activated cell sorting, as described above. Eosinophils were separated into cytoplasmic and nuclear fractions using the Nuclear and Cytoplasmic Extraction Kit (Thermo Scientific). Nuclear and cytoplasmic fractions were resolved by SDS-polyacrylamide gel electrophoresis and probed by western blotting using the antibodies listed in Table 3. The total cell extracts were prepared using RIPA buffer (50mM Tris pH 8.0, 150 mM Nacl, 1% NP40, 0.25% sodium deoxycholate, 0.1% SDS, 2mM EDTA) supplemented with 1X protease inhibitor cocktail (Thermo Fisher).

**Table 3:** Antibodies used for Western blotting.

Antibody	Manufacturer/Catalog no.
RORγt	Biolegend/654302
IL-23R- rabbit polyclonal	Abcam/22059
Tata Binding protein (TBP)	Cell Signaling Technology/8515S
Tubulin	Biolegend/627901
Anti-rabbit- IgG HRP linked HRP	Cell Signaling Technology/7077S
Anti-mouse IgG- HRP linked Ab	Cell Signaling Technology/7076
Anti-rat IgG- HRP linked Ab	Cell Signaling Technology/7074

### RT-PCR and Quantitative Real-Time RT-PCR

Total RNA was isolated using the TRIzol (Thermo Fisher) extraction method, as per the manufacturer’s instructions. RNA was reverse-transcribed using Protoscript II first strand cDNA synthesis kit (New England Biolabs). The synthesized cDNA was used to study *RORγt*, *IL-17A*, *IL-23R* gene expression using described primers [60]. *Gapdh* was used as a loading control. RT-PCR primers sequences were as follows: IL-17A-Fw, 5’-CTCCAGAAGGCCCTCAGAC-3’; IL-17A-Rv, 5’-AGCTTTCCCTCCGCATTGACACAG-3’; RORγt-Fw, 5’- CGCTGAGAGGGCTTCAC-3’; RORγt-Rv, 5’- TGCAGGAGTAGGCCACA-3’; GAPDH-Fw, 5’- AGTATGACTCCACTCACGGCAA-3’; GAPDH-Rv, 5’-TCTCGCTCCTGGAAGATGGT-3’, IL-23R-Fw, 5’-GCTCGGATTTGGTATAAAGG-3’; IL-23R-Rv, 5’-ACTTGGTATCTATGTAGGTAGG-3’. Quantitative RT-PCR was performed with SYBR master mix (Applied Biosystem) using Quant Studio-3 (Applied Biosystem). Relative gene expression was analyzed using the 2^-ΔΔ*CT*^ method [61]. *Gapdh* was used as the internal control. All genes were tested in triplicate.

### Statistical analysis

GraphPad Prism 7 software was used for statistical analysis. The unpaired two-sample Student’s t-test was used to compare means of two groups. The Bonferroni correction was applied for multiple comparisons.

## Supporting information

S1 Fig Eosinophil gating strategy. Total lung cells were prepared from mice with allergic aspergillosis using the MACS lung dissociation kit (Miltenyi Biotec). Following staining, cells were analyzed by flow cytometry. Eosinophils were defined based on the sequential gating strategy shown in the figure. FSC, forward scatter. SSC, side scatter.
(DOCX)

S2 Fig Immunoblotting for RORγt in thymic cells from wild-type and RORγt^-/-^ mice. Thymic cells were isolated from thymuses harvested from wild-type (WT) and RORγt^-/-^ (KO) mice. Cytoplasmic and nuclear extracts were prepared by cellular fractionation, resolved by SDS-PAGE, and analyzed by immunoblotting with an antibody directed against RORγt. Migration of the molecular size markers (in kDa) is shown on the right. The expected size of the RORγt band is 58 kDa. The immunoblot is representative of 2 separate experiments.
(DOCX)

S3 Fig Quantification of confocal microscopy data. Mice were challenged with *A*. *fumigatus* conidia as described in Fig 2D. BAL cells were permeabilized and stained for RORγt and the eosinophil marker MBP. A total of 205 cells in 25 microscope fields were analyzed by confocal microscopy for the presence of RORγt and MBP. Data are expressed as mean (± SE) percentage of cells staining for the indicated combination of the two markers.
(DOCX)

S4 Fig RORγt gene expression in thymic mRNA from wild type and RORc^-/-^ mice. Total thymic mRNA from wild type and RORc^-/-^ mice were analyzed for *RORγt* expression. *Gapdh* was monitored as a loading control. M: 100 bp DNA marker. Each lane shows results from an individual mouse.
(DOCX)

S5 Fig Intracellular expression of RORγt in lung eosinophils and CD4^+^ T cells from wild-type versus IL-23p19^-/-^ mice with allergic aspergillosis. Allergic aspergillosis was induced in wild-type and IL-23p19^-/-^ mice as described in Figs 1A and 6A. Upper panel: Gated lung eosinophils were analyzed for RORγt staining by flow cytometry with ICS. Lower panel: Total lung CD4^+^ T cells were gated and analyzed for RORγt expression. Representative histograms are shown.
(DOCX)

S6 Fig Pulmonary eosinophils comparing wild-type and IL-23p19^-/-^ mice with allergic aspergillosis. Allergic aspergillosis was induced in IL-23p19^-/-^ and wild-type mice as described in Fig 1A. Two days after the 3^rd^ intranasal challenge, mice were euthanized, and single cells lung suspensions were prepared. The left panel (A) shows the percentage of singlet, live, and CD45 positive cells in the lungs which were eosinophils. The right panel (B) shows the absolute numbers of eosinophils per lung. Eosinophil numbers were quantified by counting total nucleated lung cells on a hemocytometer and then multiplying by the fraction of the total cells that were eosinophils as determined by flow cytometry (singlet^+^, live^+^, CD45^+^, SiglecF^+^, and CD11c^-, low, intermediate^). See Figs 1B and 2A, and the Supplemental Reference for details. The data are expressed as means + SE (n = 6 mice per group, from two independent experiments).
(DOCX)
