# Supplementary material for: Lung eosinophils elicited during allergic and acute aspergillosis express RORγt and IL-23R but do not require IL-23 for IL-17 production
Source: PLoS Pathog. 2021 Aug 31;17(8):e1009891. doi: 10.1371/journal.ppat.1009891 (PMC8437264; doi:10.1371/journal.ppat.1009891)
Supplement: S1 Fig — Total lung cells were prepared from mice with allergic aspergillosis using the MACS lung dissociation kit (Miltenyi Biotec). Following staining, cells were analyzed by flow cytometry. Eosinophils were defined based on the sequential gating strategy shown in the figure. FSC, forward scatter. SSC, side scatter. (DOCX) [file ppat.1009891.s001.docx]

**S1 Fig. *Eosinophil gating strategy***


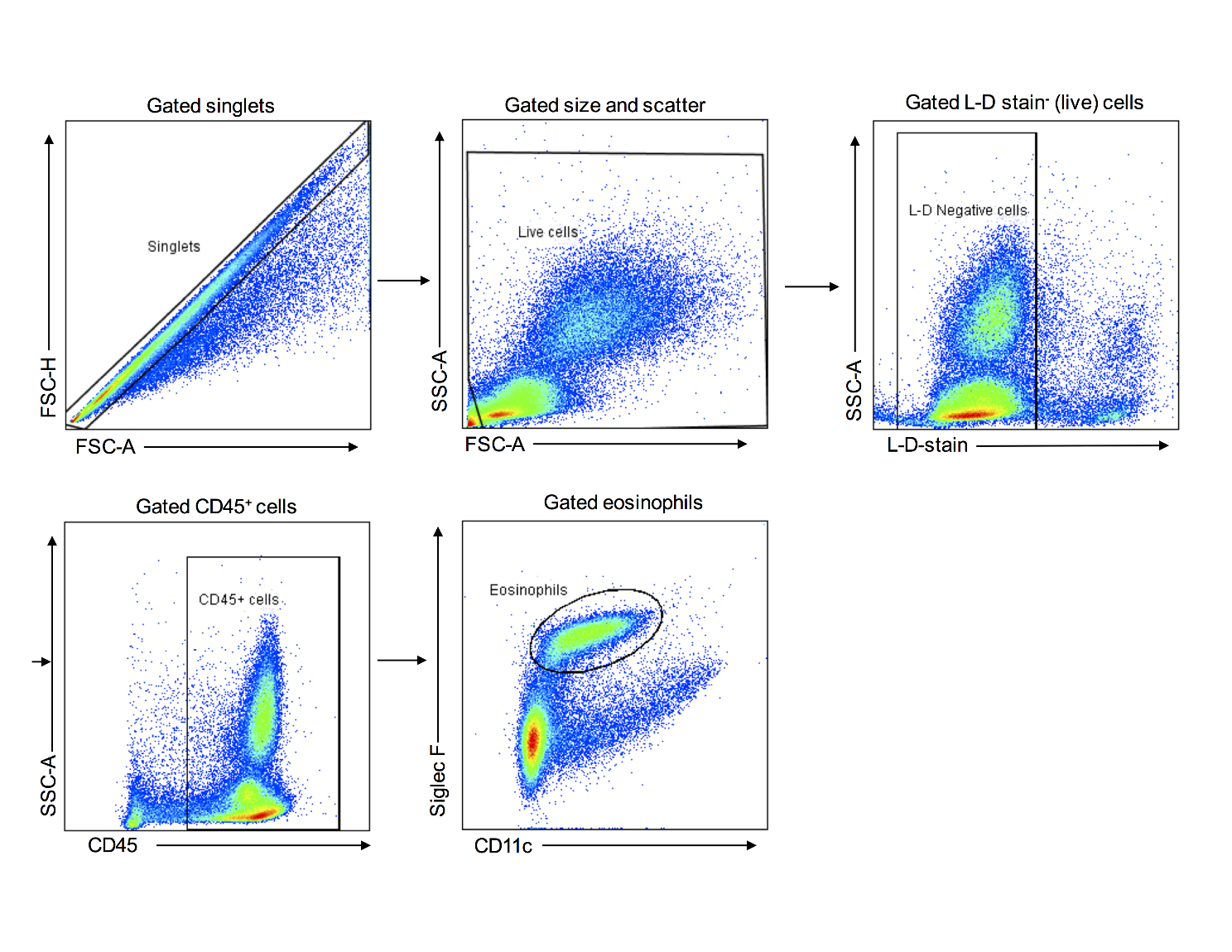


Total lung cells were prepared from mice with allergic aspergillosis using the MACS lung dissociation kit (Miltenyi Biotec). Following staining, cells were analyzed by flow cytometry. Eosinophils were defined based on the sequential gating strategy shown in the figure. FSC, forward scatter. SSC, side scatter.
