# Supplementary material for: Lung eosinophils elicited during allergic and acute aspergillosis express RORγt and IL-23R but do not require IL-23 for IL-17 production
Source: PLoS Pathog. 2021 Aug 31;17(8):e1009891. doi: 10.1371/journal.ppat.1009891 (PMC8437264; doi:10.1371/journal.ppat.1009891)
Supplement: S2 Fig — Thymic cells were isolated from thymuses harvested from wild-type (WT) and RORγt-/- (KO) mice. Cytoplasmic and nuclear extracts were prepared by cellular fractionation, resolved by SDS-PAGE, and analyzed by immunoblotting with an antibody directed against RORγt. Migration of the molecular size markers (in kDa) is shown on the right. The expected size of the RORγt band is 58 kDa. The immunoblot is representative of 2 separate experiments. (DOCX) [file ppat.1009891.s002.docx]

**S2 Fig. *Immunoblotting for ROR*ɣ*t in thymic cells from wild-type and ROR*ɣ*t^-/-^ mice.***


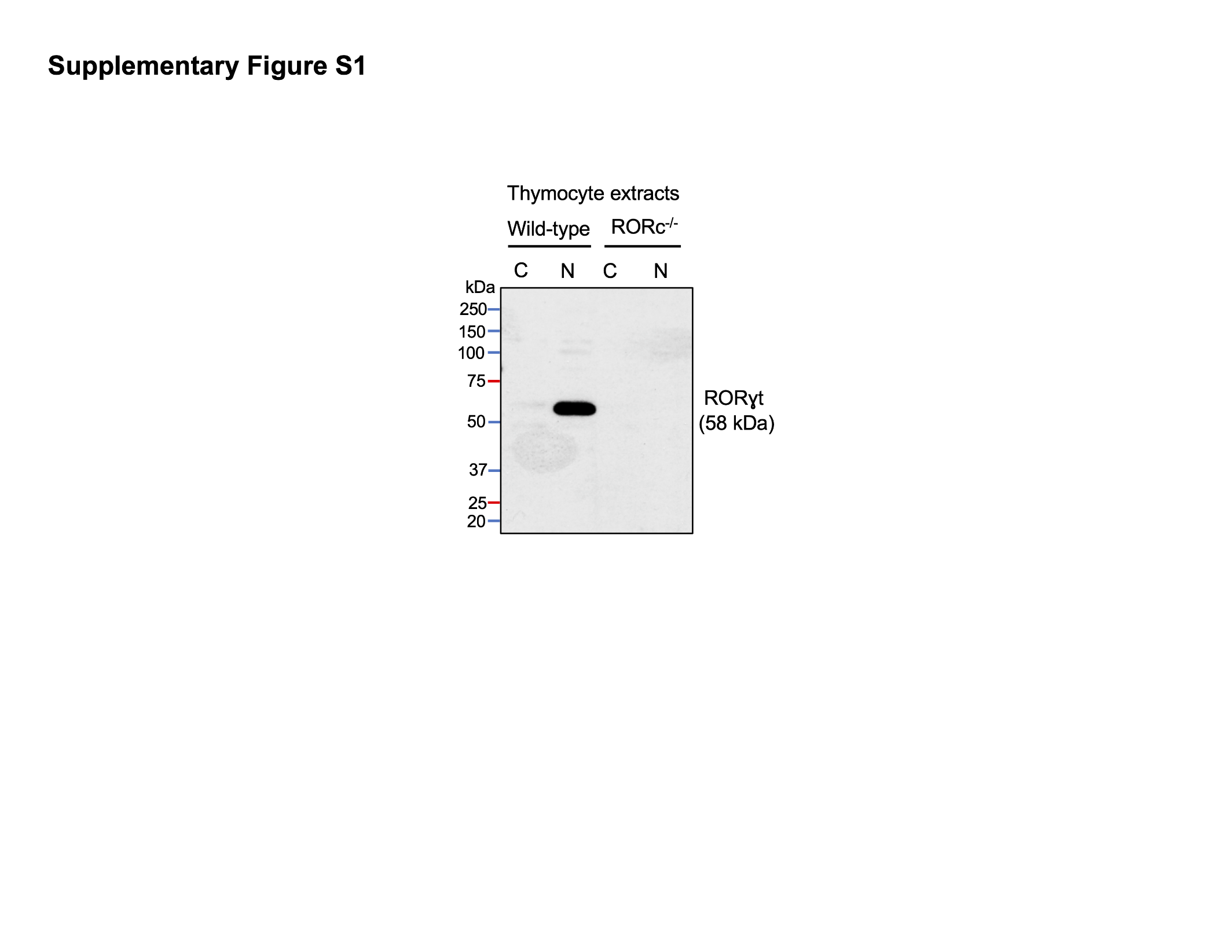


Thymic cells were isolated from thymuses harvested from wild-type (WT) and RORɣt^-/-^ (KO) mice. Cytoplasmic and nuclear extracts were prepared by cellular fractionation, resolved by SDS-PAGE, and analyzed by immunoblotting with an antibody directed against RORɣt. Migration of the molecular size markers (in kDa) is shown on the right. The expected size of the RORɣt band is 58 kDa. The immunoblot is representative of 2 separate experiments.
