# Supplementary material for: Lung eosinophils elicited during allergic and acute aspergillosis express RORγt and IL-23R but do not require IL-23 for IL-17 production
Source: PLoS Pathog. 2021 Aug 31;17(8):e1009891. doi: 10.1371/journal.ppat.1009891 (PMC8437264; doi:10.1371/journal.ppat.1009891)
Supplement: S3 Fig — Mice were challenged with A. fumigatus conidia as described in Fig 2D. BAL cells were permeabilized and stained for RORγt and the eosinophil marker MBP. A total of 205 cells in 25 microscope fields were analyzed by confocal microscopy for the presence of RORγt and MBP. Data are expressed as mean (± SE) percentage of cells staining for the indicated combination of the two markers. (DOCX) [file ppat.1009891.s003.docx]

**S3 Fig.** ***Quantification of confocal microscopy data.***

% counted cells

Mice were challenged with *A. fumigatus* conidia as described in Fig 2D. BAL cells were permeabilized and stained for RORɣt and the eosinophil marker MBP. A total of 205 cells in 25 microscope fields were analyzed by confocal microscopy for the presence of RORɣt and MBP. Data are expressed as mean (± SE) percentage of cells staining for the indicated combination of the two markers.
