# Supplementary material for: Lung eosinophils elicited during allergic and acute aspergillosis express RORγt and IL-23R but do not require IL-23 for IL-17 production
Source: PLoS Pathog. 2021 Aug 31;17(8):e1009891. doi: 10.1371/journal.ppat.1009891 (PMC8437264; doi:10.1371/journal.ppat.1009891)
Supplement: S4 Fig — Total thymic mRNA from wild type and RORc-/- mice were analyzed for RORγt expression. Gapdh was monitored as a loading control. M: 100 bp DNA marker. Each lane shows results from an individual mouse. (DOCX) [file ppat.1009891.s004.docx]

**S4 Fig. *RORɣt gene expression in thymic mRNA from wild type and RORc^-/-^ mice.***


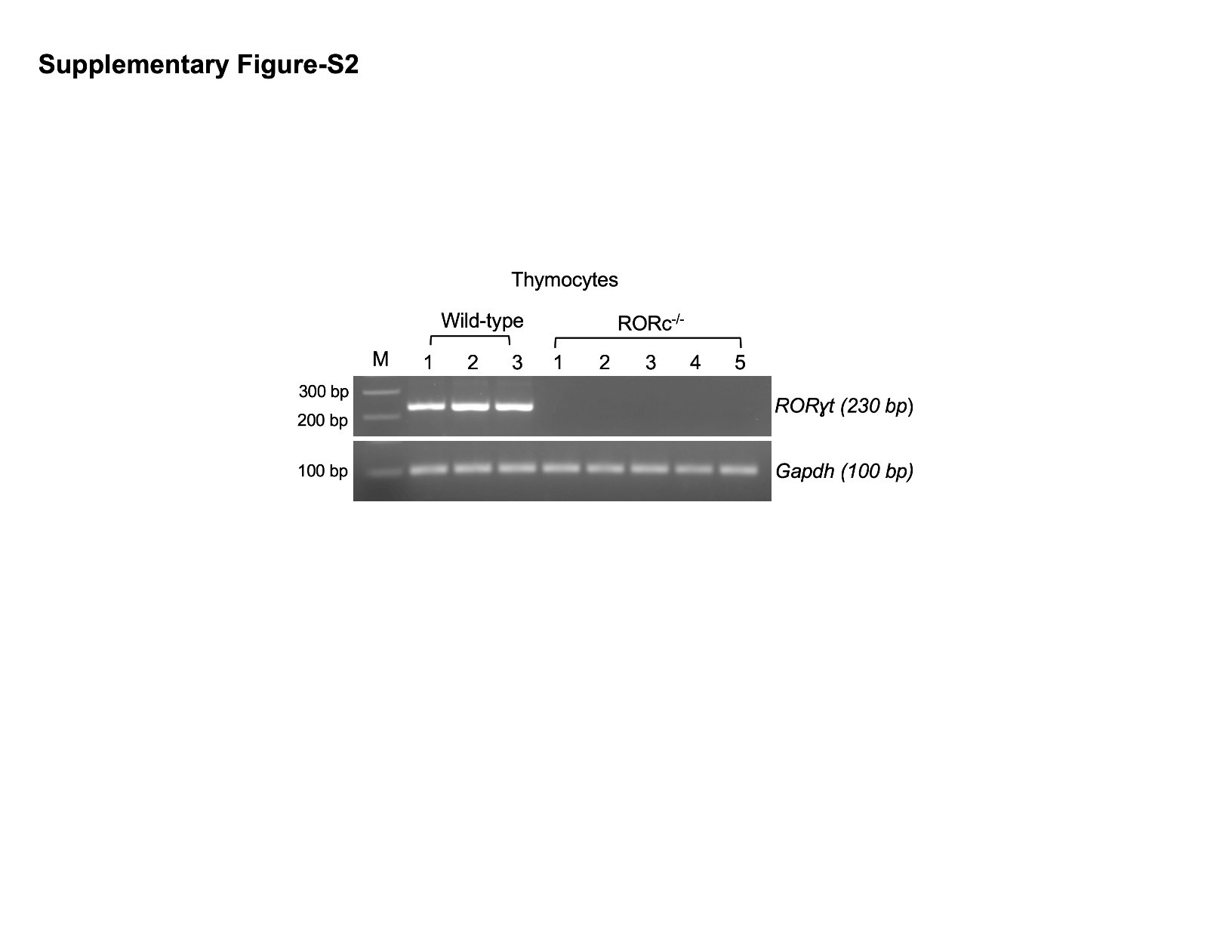


Total thymic mRNA from wild type and RORc^-/-^ mice were analyzed for *RORɣt* expression. *Gapdh* was monitored as a loading control. M: 100 bp DNA marker. Each lane shows results from an individual mouse.
