# Supplementary material for: Lung eosinophils elicited during allergic and acute aspergillosis express RORγt and IL-23R but do not require IL-23 for IL-17 production
Source: PLoS Pathog. 2021 Aug 31;17(8):e1009891. doi: 10.1371/journal.ppat.1009891 (PMC8437264; doi:10.1371/journal.ppat.1009891)
Supplement: S5 Fig — Allergic aspergillosis was induced in wild-type and IL-23p19-/- mice as described in Figs 1A and 6A. Upper panel: Gated lung eosinophils were analyzed for RORγt staining by flow cytometry with ICS. Lower panel: Total lung CD4+ T cells were gated and analyzed for RORγt expression. Representative histograms are shown. (DOCX) [file ppat.1009891.s005.docx]

**S5 Fig. *Intracellular expression of RORɣt in lung eosinophils and CD4^+^ T cells from wild-type versus IL-23p19^-/-^ mice with allergic aspergillosis***


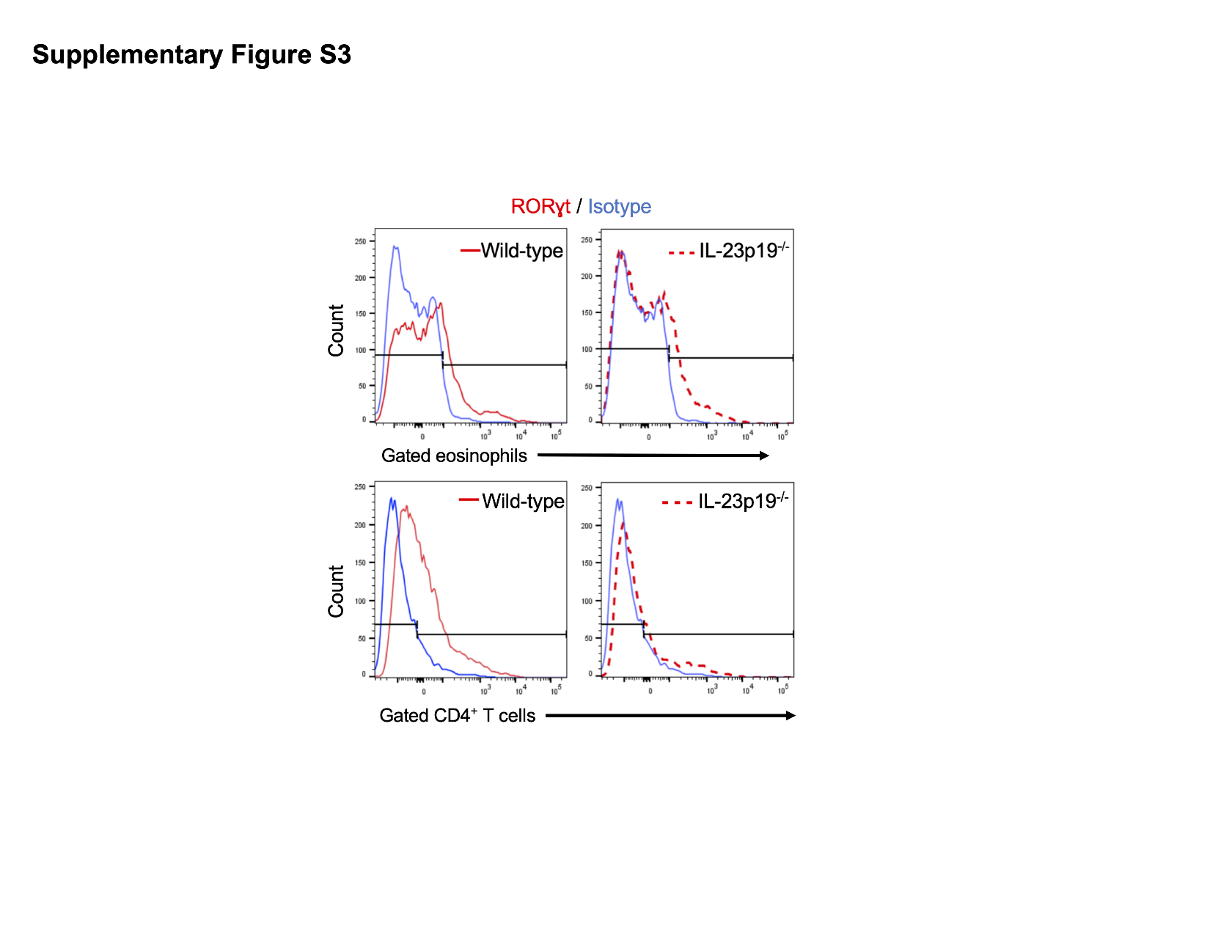


Allergic aspergillosis was induced in wild-type and IL-23p19^-/-^ mice as described in Figs 1A and 6A. Upper panel: Gated lung eosinophils were analyzed for RORɣt staining by flow cytometry with ICS. Lower panel: Total lung CD4^+^ T cells were gated and analyzed for RORɣt expression. Representative histograms are shown.
