# Supplementary material for: Lung eosinophils elicited during allergic and acute aspergillosis express RORγt and IL-23R but do not require IL-23 for IL-17 production
Source: PLoS Pathog. 2021 Aug 31;17(8):e1009891. doi: 10.1371/journal.ppat.1009891 (PMC8437264; doi:10.1371/journal.ppat.1009891)
Supplement: S6 Fig — Allergic aspergillosis was induced in IL-23p19-/- and wild-type mice as described in Fig 1A. Two days after the 3rd intranasal challenge, mice were euthanized, and single cells lung suspensions were prepared. The left panel (A) shows the percentage of singlet, live, and CD45 positive cells in the lungs which were eosinophils. The right panel (B) shows the absolute numbers of eosinophils per lung. Eosinophil numbers were quantified by counting total nucleated lung cells on a hemocytometer and then multiplying by the fraction of the total cells that were eosinophils as determined by flow cytometry (singlet+, live+, CD45+, SiglecF+, and CD11c-, low, intermediate). See Figs 1B and 2A, and the Supplemental Reference for details. The data are expressed as means + SE (n = 6 mice per group, from two independent experiments). (DOCX) [file ppat.1009891.s006.docx]

**S6 Fig. *Pulmonary eosinophils comparing wild-type and IL-23p19^-/-^ mice with allergic aspergillosis.***

Allergic aspergillosis was induced in IL-23p19^-/-^ and wild-type mice as described in Fig 1A.**
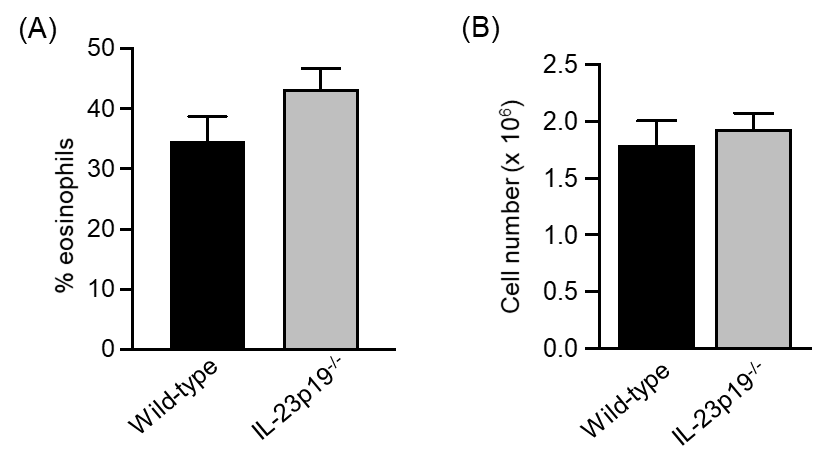
** Two days after the 3^rd^ intranasal challenge, mice were euthanized, and single cells lung suspensions were prepared. The left panel (A) shows the percentage of singlet, live, and CD45 positive cells in the lungs which were eosinophils. The right panel (B) shows the absolute numbers of eosinophils per lung. Eosinophil numbers were quantified by counting total nucleated lung cells on a hemocytometer and then multiplying by the fraction of the total cells that were eosinophils as determined by flow cytometry (singlet^+^, live^+^, CD45^+^, SiglecF^+^, and CD11c^-, low, intermediate^). See Figs 1B and 2A, and the Supplemental Reference for details. The data are expressed as means + SE (n= 6 mice per group, from two independent experiments).

Supplemental Reference. Mesnil et al. Lung-resident eosinophils represent a distinct regulatory eosinophil subset. J Clin Invest. 2016; 126:3279-95.
